# Draft genome sequence of a representative strain of the *Catenibacterium* genus isolated from human feces

**DOI:** 10.1128/MRA.00329-23

**Published:** 2023-07-26

**Authors:** Liviana Ricci, Marta Selma-Royo, Davide Golzato, Amir Nabinejad, Charlotte Servais, Federica Armanini, Francesco Asnicar, Federica Pinto, Sabrina Tamburini, Nicola Segata

**Affiliations:** 1 Department CIBIO, University of Trento, Trento, Italy; 2 IEO, European Institute of Oncology IRCCS, Milan, Italy; Queens College Department of Biology, Queens, New York, USA

**Keywords:** Catenibacterium, gut microbiome

## Abstract

A strain from a previously undescribed species belonging to the *Catenibacterium* genus was isolated from the stool of a healthy volunteer. The strain is strictly anaerobic, and the genome encodes a CRISPR-Cas system and genes related to trimethylamine production.

## ANNOUNCEMENT

The *Catenibacterium* genus belongs to the Erysipelotrichaceae family, with only one known species, *Catenibacterium mitsuokai* (1). Metagenome-assembled genomes suggest an expanded predicted diversity for the *Catenibacterium* genus (2), which also comprises some taxonomically unassigned isolates.

*Catenibacterium* strain CMD8551 was isolated in an anaerobic chamber (95% N₂/5% H₂) from the feces of a healthy volunteer (protocol no. 2021–007 by the Ethical Committee of the University of Trento). The fresh sample was homogenized, serially diluted, and spread (10^−5^ dilution) onto chopped meat agar plates with 4% defibrinated sheep blood (Microbiol Diagnostics), 0.2% carbon sources (pectin, arabinoxylan, glucose, maltose, cellobiose, and resistant starch), 2.5 g/L yeast extract, 0.005% vitamin K1, and 5 mg/L hemin. After 2 days at 37°C, a single colony was inoculated in modified chopped meat broth.

Genomic DNA was isolated from liquid culture using the Wizard Genomic DNA Purification Kit (Promega) and used for library preparation with Illumina DNA Prep and Tagmentation Kit (Illumina). Libraries were sequenced (150 bp paired-end reads) on a NovaSeq6000 S4 flowcell (Illumina) at the University of Trento, following a cleaning step (0.6× Agencourt AMPure XP beads). Raw reads were filtered with Trim Galore (parameters: --stringency 5 --length 75 --quality 20 --max_n 2 --trim-n; https://github.com/FelixKrueger/TrimGalore). Illumina PhiX adapters and human DNA reads were removed using Bowtie2 (3) against the corresponding reference genomes. A total of 23,527,050 high-quality paired-end reads (mean Q value of 35.70) were retained (mean read length of 147.48 bp). Genome assembly was performed using SPAdes 3.15.2 (parameters: --careful -k 21,33,55,77,99,127) (4) on 40% randomly rarefied reads (https://github.com/lh3/seqtk, parameters: sample module). Assembly statistics, computed using QUAST v5.1.0rc1 (5), completeness, and contamination obtained with CheckM v1.1.2 (6), are reported in Table 1. Mean coverage was computed using CMseq v1.0.4 (https://github.com/SegataLab/cmseq). Unless specified, all the computational tools used in this work were applied with default parameters.

**TABLE 1:** Summary of the statistics from *Catenibacterium* CMD8551 genome assembly

Parameter	Value
Total length (bp)	2,320,430
No. of scaffolds	113
GC content (%)	33.7
Mean coverage (×)	519×
Size of longest scaffold (bp)	105,579
*N*_50_ (bp)	44,791
*L*_50_	18
No. of coding sequences	2,239
No. of RNAs	10 rRNA 76 tRNA
Estimated completeness (%)	100%
Estimated contamination (%)	0%

The Prokka (v1.14) (7) annotation of the assembly revealed a total of 2,326 genes, including CRISPR-Cas system and genes for choline metabolism (8). Fifteen glycoside hydrolases were identified by dbCAN2, suggesting high carbohydrate catabolic potential. The analysis with *Resistance Gene Identifier* (version 5.1.1) (9) showed four putative genes involved in resistance to carbapenem and glycopeptide antibiotics.

**Fig 1:**
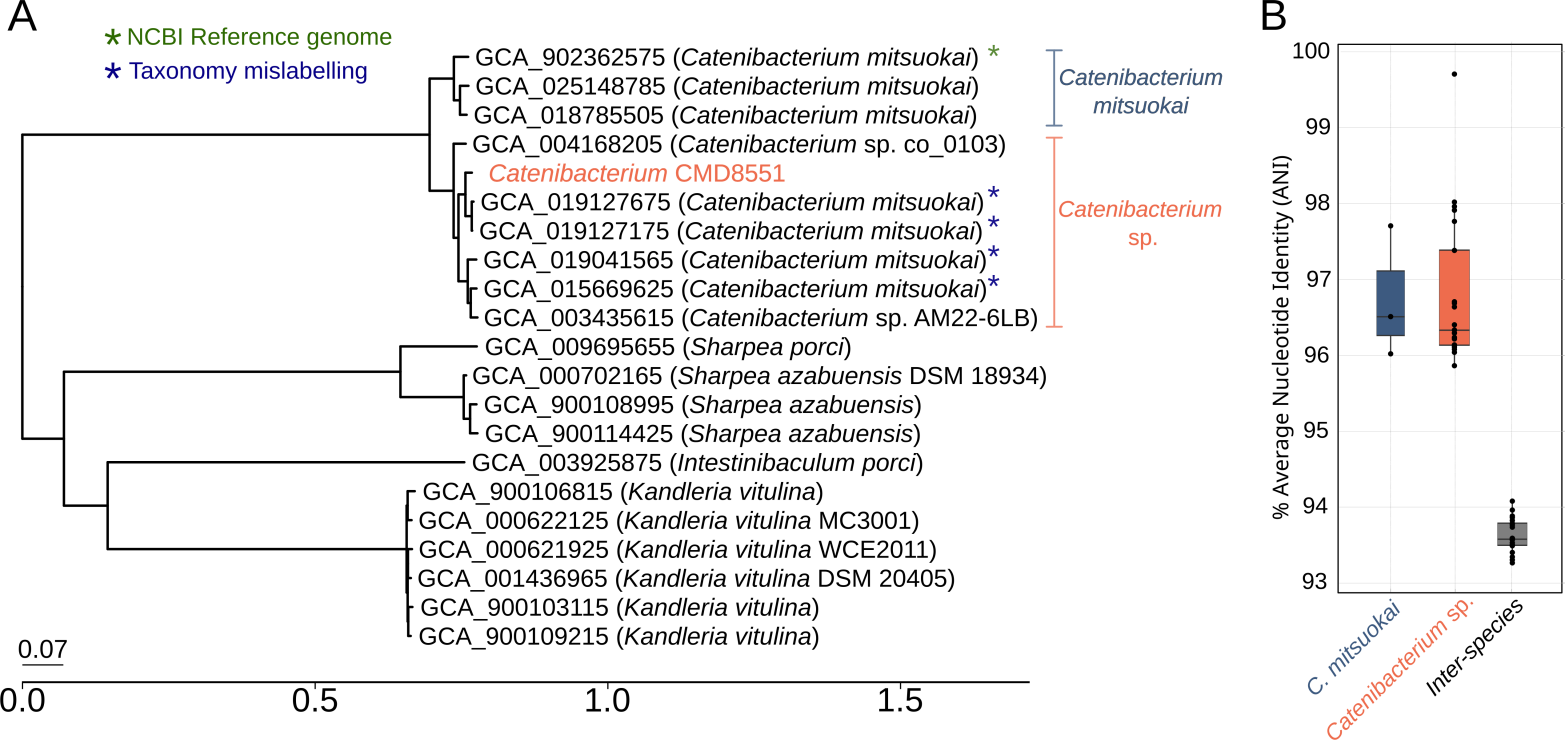
(**A**) Phylogenetic tree of the new *Catenibacterium* isolate and related taxa with available reference genomes obtained from sequencing of isolates. The marked reference genomes were taxonomical missasigned (as *C. mitsuokai*) based on average nucleotide identity (ANI, <94%) to its reference strain (GCA_902362575). These reference genomes, along with the CMD8551 strain, cluster as a different taxonomic group distant from *C. mitsuokai* compatible with a new species inside the *Catenibacterium* genus. (**B**) Comparison of the ANI of the available genomes from *Catenibacterium* genus inside each species genomes defined by the phylogenetic tree (*C. mitsuokai* and the proposed *C. tridentinum*) and between them (interspecies).

PhyloPhlAn version 3.0 (January 21 database version) (10) was used to perform taxonomic assignment and reconstruct the phylogenetic tree in Fig. 1 (parameters: -d phylophlan --diversity low --fast --force_nucleotides), using MAFFT (v7.508) for the multiple-sequence alignment step of the 400 universal proteins (11) and RAxML (v8.2.12) for the phylogenetic reconstruction (GTR + CAT substitution model). *C. mitsuokai* (GCA_018785505) was the closest species to the new strain, with three reference genomes showing <94% average nucleotide identity (ANI) values (Fig. 1). By comparing the strain assembly with NCBI’s publicly available reference genomes assigned to *C. mitsuokai*, two subtrees were clearly identified, corresponding to *C. mitsuokai* and a previously unidentified species. We propose the species name *Catenibacterium tridentinum,* represented by the strain CMD8551, and the other genomes previously assigned to *C. mitsuokai* that show species-level similarity (intraspecies min–max ANI%: 95.85–99.77).

## Data Availability

This study project is available under NCBI accession ID PRJNA939950. The sample used to assemble the genome and the assembly areisare available under accession SAMN33794588 and JARNBK000000000, respectively.

## References

[1] Kageyama A , Benno Y . 2000. Catenibacterium Mitsuokai gen. nov., sp. nov., a gram-positive anaerobic bacterium isolated from human faeces. Int J Syst Evol Microbiol 50 Pt 4:1595–1599. doi:10.1099/00207713-50-4-1595 10939666

[2] Blanco-Míguez A , Beghini F , Cumbo F , McIver LJ , Thompson KN , Zolfo M , Manghi P , Dubois L , Huang KD , Thomas AM , Nickols WA , Piccinno G , Piperni E , Punčochář M , Valles-Colomer M , Tett A , Giordano F , Davies R , Wolf J , Berry SE , Spector TD , Franzosa EA , Pasolli E , Asnicar F , Huttenhower C , Segata N . 2023. Extending and improving metagenomic taxonomic profiling with uncharacterized species using MetaPhlAn 4. Nat Biotechnol. doi:10.1038/s41587-023-01688-w PMC10635831 36823356

[3] Langmead B , Salzberg SL . 2012. Fast gapped-read alignment with Bowtie 2. Nat Methods 9:357–359. doi:10.1038/nmeth.1923 22388286 PMC3322381

[4] Bankevich A , Nurk S , Antipov D , Gurevich AA , Dvorkin M , Kulikov AS , Lesin VM , Nikolenko SI , Pham S , Prjibelski AD , Pyshkin AV , Sirotkin AV , Vyahhi N , Tesler G , Alekseyev MA , Pevzner PA . 2012. Spades: a new genome assembly algorithm and its applications to single-cell sequencing. J Comput Biol 19:455–477. doi:10.1089/cmb.2012.0021 22506599 PMC3342519

[5] Mikheenko A , Prjibelski A , Saveliev V , Antipov D , Gurevich A . 2018. Versatile genome assembly evaluation with QUAST-LG. Bioinformatics 34:i142–i150. doi:10.1093/bioinformatics/bty266 29949969 PMC6022658

[6] Parks DH , Imelfort M , Skennerton CT , Hugenholtz P , Tyson GW . 2015. Checkm: assessing the quality of microbial genomes recovered from isolates, single cells, and metagenomes. Genome Res 25:1043–1055. doi:10.1101/gr.186072.114 25977477 PMC4484387

[7] Seemann T . 2014. Prokka: rapid prokaryotic genome annotation. Bioinformatics 30:2068–2069. doi:10.1093/bioinformatics/btu153 24642063

[8] Rath S , Heidrich B , Pieper DH , Vital M . 2017. Uncovering the trimethylamine-producing bacteria of the human gut microbiota. Microbiome 5:54. doi:10.1186/s40168-017-0271-9 28506279 PMC5433236

[9] Alcock BP , Raphenya AR , Lau TTY , Tsang KK , Bouchard M , Edalatmand A , Huynh W , Nguyen A-L , Cheng AA , Liu S , Min SY , Miroshnichenko A , Tran H-K , Werfalli RE , Nasir JA , Oloni M , Speicher DJ , Florescu A , Singh B , Faltyn M , Hernandez-Koutoucheva A , Sharma AN , Bordeleau E , Pawlowski AC , Zubyk HL , Dooley D , Griffiths E , Maguire F , Winsor GL , Beiko RG , Brinkman FSL , Hsiao WWL , Domselaar GV , McArthur AG . 2020. CARD 2020: antibiotic resistome surveillance with the comprehensive antibiotic resistance database. Nucleic Acids Res 48:D517–D525. doi:10.1093/nar/gkz935 31665441 PMC7145624

[10] Asnicar F , Thomas AM , Beghini F , Mengoni C , Manara S , Manghi P , Zhu Q , Bolzan M , Cumbo F , May U , Sanders JG , Zolfo M , Kopylova E , Pasolli E , Knight R , Mirarab S , Huttenhower C , Segata N . 2020. Precise phylogenetic analysis of microbial isolates and genomes from metagenomes using PhyloPhlAn 3.0. Nat Commun 11:2500. doi:10.1038/s41467-020-16366-7 32427907 PMC7237447

[11] Segata N , Börnigen D , Morgan XC , Huttenhower C . 2013. Phylophlan is a new method for improved phylogenetic and taxonomic placement of microbes. Nat Commun 4:2304. doi:10.1038/ncomms3304 23942190 PMC3760377

